# Posterior Wall Involvement During Pulmonary Vein Isolation Using the Farapulse System

**DOI:** 10.1002/joa3.70171

**Published:** 2025-08-07

**Authors:** Maria Teresa Izquierdo de Francisco, Josep Navarro‐Manchon, Oscar Cano Perez, Javier Navarrete Navarro, Carmen Arveras Martinez, Fransciso Javier Chorro Gasco, Luis Martinez‐Dolz, Joaquin Osca Asensi

**Affiliations:** ^1^ Electrophysiology Section, Cardiology Department Hospital Universitari i Politecnic La Fe Valencia Spain; ^2^ Instituto de Investigación Sanitaria La Fe (IIS La Fe) Valencia Spain; ^3^ Universitat Valencia Medicine Department Valencia Spain

**Keywords:** posterior wall, pulmonar vein isolation, pulsed field ablation

## Abstract

**Background:**

First approved PFA (Pulsed‐Field‐Ablation) system for pulmonary vein isolation (PVI) has been Farapulse PFA system. The aim was to assess the characteristics of the lesion made by the Farapulse system and its influence on the clinical results.

**Methods:**

First 76 consecutive patients referred for PVI and treated with the Farapulse PFA system were included. A voltage and an activation map were performed before and after PVI. An imaginary middle line was measured between the two carinas. Fusion on the posterior wall was defined when the contralateral ablation areas were connected. We arbitrarily defined a narrow corridor as one that measured < 20 mm of healthy tissue (voltage > 0.5 mV).

**Results:**

Post‐PVI mapping revealed an unexpected narrow corridor in the posterior wall in 12 (15%) and fusion in 18 (23%) patients. The multivariate analysis revealed that the only independent predictor was the length of the middle inter‐carinas line. The length of the middle posterior line was significantly shorter in patients with affectation of the posterior wall (62 ± 2 vs. 71 ± 3 mm, *p* = 0.0001). ROC curve showed that a middle line cutoff value of 65 mm offered a sensitivity and specificity of 80% and 70% (AUC: 0.82; 95% CI: 0.59–0.84). A corridor < 10 mm is associated with slow conduction velocity below 0.7 m/s, but narrow corridor or fusion were not associated with atrial fibrillation recurrences.

**Conclusions:**

30 (40%) patients showed narrow corridor or fusion on the posterior wall. The only independent predictor was the length of the middle inter‐carina line.

## Graphical abstract

Post‐PVI mapping revealed an unexpected narrow corridor in the posterior wall in 12 (15%) and fusion in 18 (23%) patients. The multivariate analysis revealed that the only independent predictor was the length of the middle inter‐carina line. A corridor < 10 mm is associated with slow conduction velocity below 0.7 m/s, but narrow corridor or fusion were not associated with atrial fibrillation recurrences.

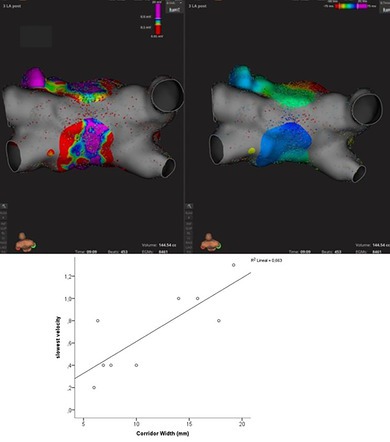

## 1 Background

Pulse field ablation (PFA) has been recently developed as a new myocardial‐specific, non‐thermal atrial fibrillation ablation method.

First approved PFA system has been the Farapulse PFA system (FARAPULSE, Boston Scientific). Farapulse system is a pentaspline basket‐like catheter that is used as a single‐shot ablation tool. However, the lesion size and shape are poorly controlled due to the fixed basket diameter (31 or 35 mm in diameter) and the entirely fluoroscopic guidance. After pulmonary veins isolation (PVI) with Farapulse, it has been observed that enlarged areas of LA isolation affected the posterior wall and the roof; even, in around 26% patients, it has been noticed that ablation lines merge at these levels when voltage maps have been created [1]; in addition, a high rate of atypical atrial flutter incidence has been reported following PVI with Farapulse, and the most frequent mechanism was the presence of a slow conduction isthmus in the posterior wall [2]. If these two facts are connected and whether excess ablation on the posterior wall favors slow conduction corridors is unknown.

The aim of this study was to assess the characteristics of the lesion made by the Farapulse system and its influence on the clinical results.

## 2 Methods

### 2.1 Patients

This study involved the first 76 consecutive patients referred for AF ablation and treated with the Farapulse system in a single center. The only exclusion criterion was the indication for ablation beyond the pulmonary veins.

The first case was performed in July 2022; all the patients signed the written informed consent before the procedure.

### 2.2 Mapping an Ablation Procedure

All the patients underwent PVI with the Farapulse PFA ablation system. As it has been previously described [3] it is a single‐shot tool that includes a PFA pentaspline catheter (Farawave) that has two ablation modes: basket‐shaped and flower‐shaped. The catheter diameter is measured in the flower‐shape configuration, and the 31 mm one was used in all cases in this study. The usual recommendations established for PVI were followed (at least 8 applications per vein: 4 with basket‐shape and 4 with flower configuration; with an energy output of 2 kV). All the procedures were performed by two experienced electrophysiologists.

All the procedures were performed with the help of the Rhythmia navigation System (Rhythmia, Boston Scientific). Each Farawave's spline contains 4 electrodes and one in each spline is available for intracardiac electrogram recording and pacing. These electrodes allow for a 3‐dimensional, non‐accurate, impedance‐based visualization in the electroanatomic navigation system.

The procedures were performed under conscious sedation and with uninterrupted oral anticoagulation. A single transseptal puncture was performed with an 8.5‐F sheath and exchanged for the 13‐F PFA sheath in the left atrium. After the first 7 cases, the transseptal puncture was performed directly with the Faradrive sheath with a 98‐cm Brockenbrough needle under fluoroscopy guidance. After the transseptal puncture, intravenous heparin was administered to achieve an activated clotting time of 350 s.

A voltage map and an activation map were performed before and after the PVI using a 64‐pole basket mapping catheter (Orion, Boston Scientific). The left atrium maps were performed either in sinus rhythm or during coronary sinus pacing. If the patient presented in atrial fibrillation, an electrical cardioversion was performed. If it was not possible to maintain sinus rhythm, the pre‐ablation mapping was performed in atrial fibrillation.

### 2.3 Map Analysis

A Left atrium bipolar voltage map was performed before and after PVI. The scar was analyzed using a cutoff of 0.1–0.5 mV as the lower and upper voltage limits.

**FIGURE 1:**
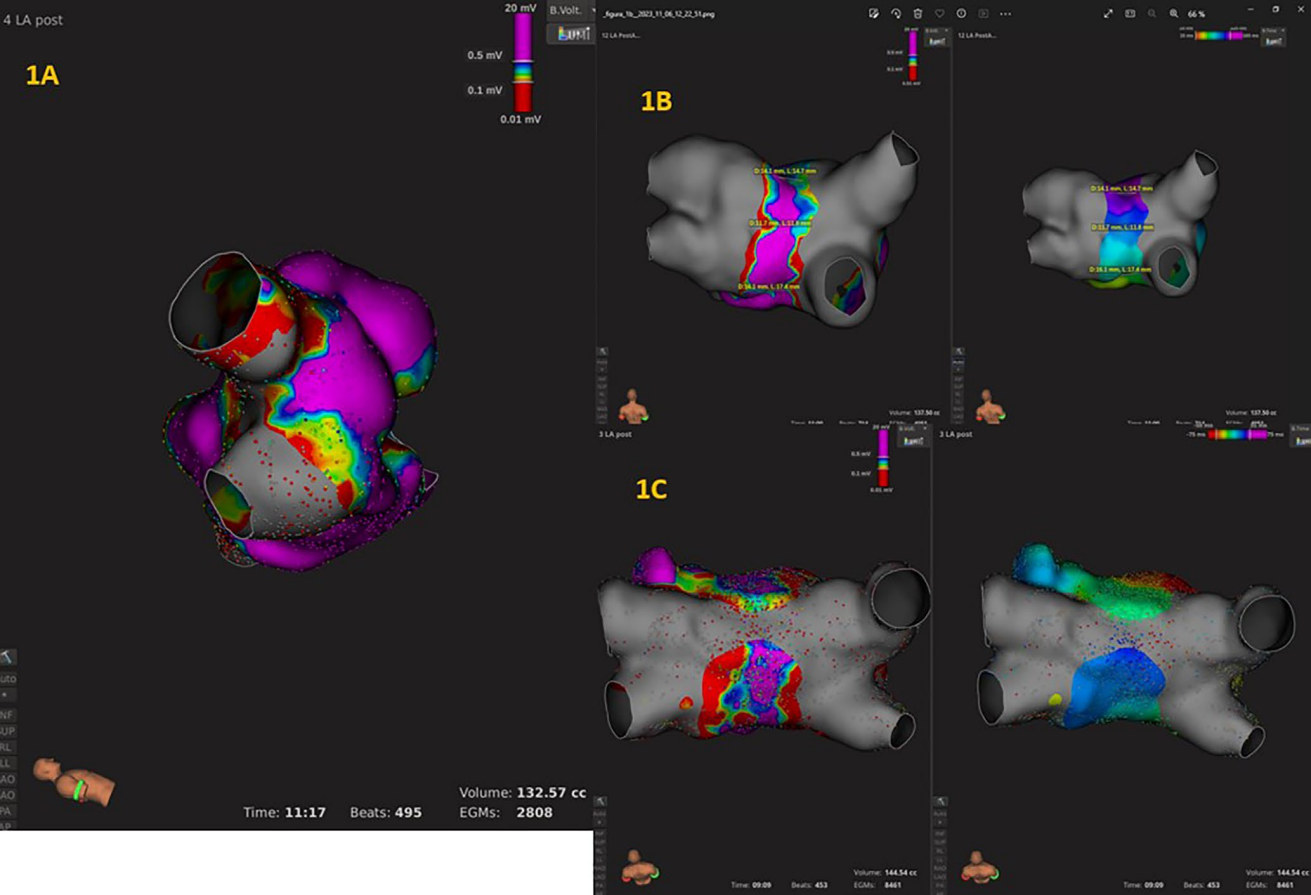
(A) In this patient, voltage map post‐PVI mapping revealed a notch of healthy tissue (> 0.5 mV) in the anterior ablation line. (B) Left: Voltage map with unexpected narrow corridor of healthy tissue (< 20 mm of tissue > 0.5 mV) in the posterior wall. Right: Velocity map. (C) Left: Voltage map with apparent fusion at the posterior wall. Right: Velocity map. This patient presented slow conduction through it.

The posterior wall surface area was defined as the area limited by two lines: the imaginary superior line that connects the superior aspect of the superior veins and the imaginary inferior line that connects the inferior aspect of the inferior veins. An imaginary middle line was measured between the two carinas (Figure 1B). The edges of the imaginary lines were positioned at the point of maximum inflection between the pulmonary vein and the left atrial wall.

We stated that there was fusion on the posterior wall when the contralateral ablation areas connected at the mid‐level of the roof or the posterior wall. We arbitrarily defined a narrow aisle on the posterior wall as one that measured < 20 mm of healthy tissue (healthy tissue was defined as that which had a voltage > 0.5 mV).

A Left atrium activation map was performed before and after PVI, but only the one after the ablation was analyzed. Isochronal activation maps were analyzed in both patients with a narrow posterior corridor (< 20 mm) and patients with fusion on the posterior wall. An isochrone was defined as the area activated during 10 ms. The width of the narrowest isochrone was measured in the posterior corridor and then the activation velocity was calculated and established as the slowest measurable speed in that area (Figure 1C). When fusion was observed, the activation map was analyzed to evaluate if there was actual conduction block or only slow conduction.

If the post‐PVI map showed a gap in any of the veins, additional applications were performed until PVI was achieved; a new post‐PVI map was created.

Despite finding an unnoticed fusion or a narrow corridor in the voltage map after PVI, no additional applications in the posterior wall were performed to completely isolate it.

### 2.4 Follow‐Up

Clinical follow‐up was conducted for all patients every 6 months. Electronic history was also reviewed in each patient to register every consultation in the emergency department.

### 2.5 Statistical Analysis

Values of categorical variables are reported as numbers and percentages, and values of continuous variables are reported as mean with standard deviation. Test of normality was conducted by Kolmogorov–Smirnov. Continuous variables were compared using Student's *t*‐test or Mann–Whitney *U* test, and categorical variables were compared using Fisher's exact or chi‐squared tests, as appropriate. Statistical significance was considered when the two‐sided *p* value was < 0.05. All analyses were performed using SPSS version 21.

All authors had full access to the data and have read and agreed to the manuscript as written. The study confirms the principles of the Declaration of Helsinki. Ethical approval was waived by the local Institutional Review Board in view of the retrospective nature of this study.

## 3 Results

### 3.1 Study Patients

Seventy‐six consecutive patients with paroxysmal AF (58%) or persistent AF (42%) referred for ablation were included in this study. Patient characteristics are shown in Table 1.

**TABLE 1:** Patients characteristics of 76 patients at baseline.

Sex	52 male (68%) 24 female (32%)
Age	60 ± 10 years
Body weight	85 ± 17 kg
Body‐mass index	29.1 ± 5 kg/m^2^
Hypertension	44 (58%)
Diabetes	14 (18%)
Dyslipidemia	40 (53%)
Smoking history	28 (37%)
Sleep apnea	13 (17%)
Previous stroke or TIA	4 Stroke (5%) 4 TIA (5%)
Atrial fibrillation	Paroxysmal 44 (58%) Persistent 38 (42%)
Previous electrical cardioversion	34 (45%)
Months since first diagnostic of atrial fibrillation	39 ± 51 months
Previous ablation	3 Pulmonary vein isolation (4%) 5 Others (7%)
Structural heart disease	56 without structural heart disease (74%) 13 dilated cardiomyopathy (17%) 3 coronary artery disease (4%) 3 hypertensive cardiopathy (4%) 1 Valve dysfunction (1%)
Medications	58 Antiarrhythmic (76%) 38 Class I (50%) 18 Class III (28%) 56 Beta‐blocker (74%) 39 ACE inhibitor or ARB (51%)
LVEF	60 Preserved (79%) 5 Mild‐reduced (7%) 7 Moderate‐reduced (9%) 4 Severe‐reduced (5%)
Left atrial planimetry	23 ± 5 cm^2^
Serum parameters	Creatinine 1 ± 0.4 mg/dL Hemoglobin 14.7 ± 1.69 g/dL NT‐pro‐BNP 934 ± 1508 pg/mL

Abbreviations: LVEF, left ventricular ejection fraction; NT‐pro‐BNP, N‐terminal pro–B‐type natriuretic peptide.

### 3.2 Ablation Procedure

PVI was achieved in 100% of the cases using the 31 mm PFA ablation catheter. Finally, 35 ± 4 applications per patient were performed on average. In 50 patients (66%), additional applications were performed beyond the eight recommended for each vein. The most common vein that received additional applications was the right superior pulmonary vein (RSPV) (9.2 ± 2.5 applications on average).

Initial post‐ablation mapping of the left atrium showed an incomplete PVI in 5 patients, 3 cases with a gap in the antero‐superior segment of the right superior pulmonary vein (RSPV), and 2 cases with a gap in the antero‐inferior segment of the left inferior pulmonary vein (LIPV). In all these cases, the PV were ablated with additional PFA applications confirming PVI in a new left atrium.

The mean procedure time was 77 ± 19 min, including pre‐ and post‐ablation mapping. The fluoroscopy time was 15 ± 6 min.

### 3.3 Voltage Map Analysis

The pre‐ablation anatomical map showed a mean left atrium volume of 144 ± 30 mL and a mean posterior wall area of 24 ± 5 cm^2^. The imaginary superior, middle, and inferior lines at the posterior wall were 48 ± 9, 68 ± 8, and 50 ± 9 mm on average. It was observed that the presence of low voltage areas (< 0.5 mV) in 22 patients.

Post‐PVI mapping revealed a notch of healthy tissue (> 0.5 mV) in the anterior ablation line, at the level of the carina in 17 patients (Figure 1A). The location of this incomplete antral ablation was the right anterior carina in 12 patients, the left anterior carina in 3 cases, and in both anterior carinas (left and right) in one case. At the posterior aspect of the antrum, the coverage was complete and homogeneous save in 1 case.

The area most affected by the ablation beyond the pulmonary veins was the posterior wall, with an ablated area covering 58% ± 19% of the posterior wall surface on average.

After PVI, it was observed that an unexpected narrow corridor of healthy tissue (< 20 mm of tissue > 0.5 mV) in the posterior wall was present in 12 patients (15%) (Figure 1B).

Based on the voltage mapping, 18 (23%) patients showed fusion of the ablation lines on the posterior wall or the roof (Figure 1C). In 17 patients, it was due to the confluence of the contralateral PV circumferential ablation lines, and in one patient because of preexisting scar zones in the roof that were added to the ablation lines. The place of the fusion was mainly at the superior (6 patients) or middle (12 patients) imaginary lines.

On the whole, 30 patients (40%) showed a narrow corridor of healthy tissue or fusion at the posterior wall.

In the univariate analysis, the variables that predicted the occurrence of a narrow corridor or fusion on the posterior wall were: a small left atrial volume and a short superior, middle, and inferior imaginary lines in the posterior wall. The multivariate analysis revealed that the only independent predictor was the length of the middle inter‐carinas line. The length of the middle posterior line was significantly shorter in patients with affectation of the posterior wall by the ablation (62 ± 2 vs. 71 ± 3 mm, *p* = 0.0001). By means of a ROC curve, it was found that a middle line cutoff value of 65 mm offered a sensitivity and specificity of 80% and 70% to identify the possibility of posterior wall affectation (AUC: 0.82; 95% CI: 0.59–0.84). On the one hand, 60% of patients with a middle posterior line below 65 mm showed an affected posterior wall; on the other hand, only 8% of patients with a distance > 65 mm showed such involvement of the posterior wall.

### 3.4 Post‐Ablation Activation Map

The post‐ablation activation map was analyzed in the 18 patients with apparent fusion at the posterior wall level in the voltage map. Of them, 16 patients showed conduction block in the activation map at the level of the fusion, and 2 patients presented slow conduction through it. In these 2 patients, the slowest activation speed measured with isochrones of 10 ms in the posterior wall was 0.4 and 0.7 m/s (Figure 1C).

**FIGURE 2:**
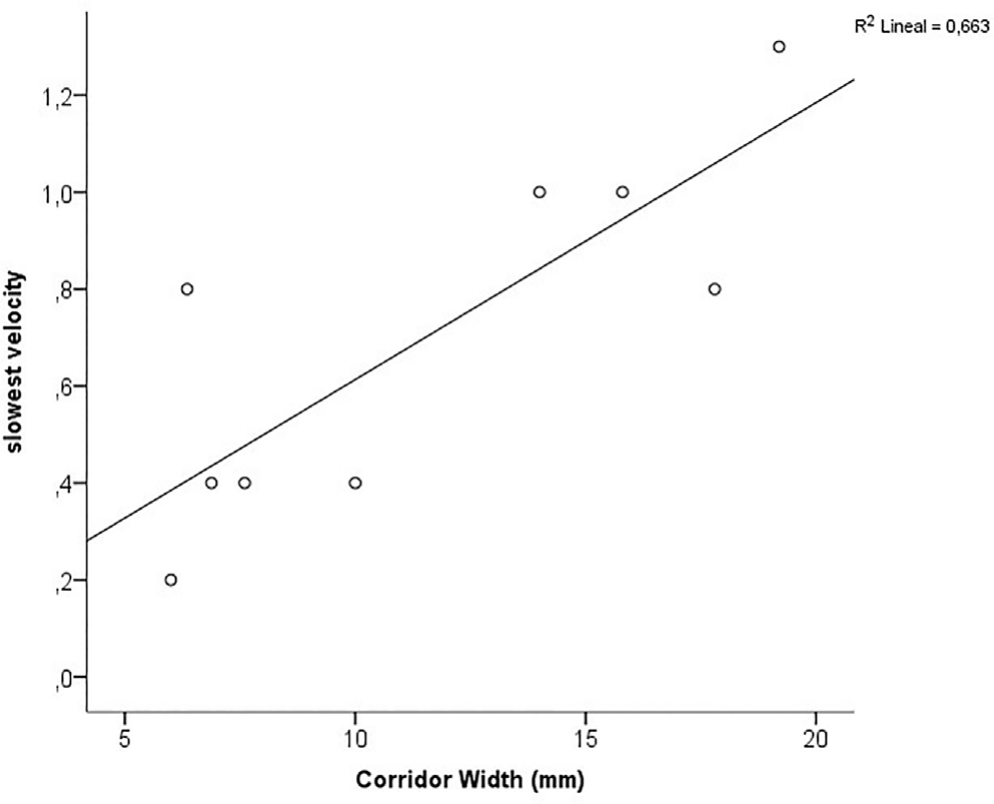
Correlation graph between the width of the posterior corridor and the slowest conduction velocity through it. The more narrow the corridor, the slower the velocity. A corridor < 10 mm is associated with a slow conduction velocity below 0.7 m/s.

The post‐ablation activation map was analyzed in 9 of the 12 patients with a narrow corridor. In the remaining 3, the activation map was unreliable due to the high density of atrial premature beats. The slowest conduction velocity through the corridor varied between 0.2 and 1.3 m/s. A strong positive correlation was found between the corridor width and the slowest conduction velocity through it (Pearson correlation coefficient = 0.8 and *R*2 = 0.66; *p* < 0.001) (Figure 2).

On the whole, 5 patients showed a velocity under 0.4 m/s through the posterior wall. A corridor < 10 mm is associated with a slow conduction velocity below 0.7 m/s (Figure 2).

### 3.5 Mid‐Term Follow Up

During the blanking period, 7 (9.2%) patients presented arrhythmia recurrence; 6 patients showed atrial fibrillation and 1 (1.3%) patient showed a self‐terminated atypical atrial flutter. This last patient had showed a narrow posterior corridor (6 mm) and slow velocity at this level (0.2 m/s) in the post‐ablation map.

Outside the blanking period, during a mean follow‐up of 360 ± 42 days, 9 (11.8%) patients experienced recurrence: 6 (7.8%) patients with atrial fibrillation, 1 (1.3%) patient with atrial fibrillation and atypical atrial flutter, and 2 (1.3%) patients with atypical atrial flutter. The two patients (2.6%) who presented with atypical atrial flutter as a recurrence had not shown either a narrow corridor or fusion in the post‐ablation map.

The presence of a narrow corridor or fusion was not associated with atrial fibrillation recurrences.

### 3.6 Safety

There were 3 complications after the procedure: one patient suffered a pericardial chest pain, one patient suffered a pericardial effusion conservatively managed, and one patient suffered a transient ischemic attack 5 days after the hospital discharge.

## 4 Discussion

This study shows the initial clinical experience in a single hospital employing the Farapulse PFA system together with the anatomical mapping system, Rhythmia, in a consecutive series of patients referred for AF ablation. The main findings were that:

1. The 31 mm‐Farawave catheter obtained a 100% acute PVI rate with a first pass non‐complete ablation in 5% of patients, mainly at anterior segments of pulmonary veins.
2. Excessive involvement of the posterior wall after PV circumferential ablation was observed, more frequently in patients with an inter‐carina distance of < 65 mm. In 18 (23%) patients, a complete ablation fusion in the posterior wall was detected in the voltage map, and 12 (15%) patients showed a post‐ablation narrow corridor < 20 mm in the posterior wall.
3. A low rate of atypical atrial flutter after PVI was noticed (3/76 patients) and only one, self‐terminated, in the blanking period, appeared in a patient who had showed a narrow corridor in the posterior wall in the post‐PVI map.

These results show that the Farapulse PFA ablation system was associated with a 100% acute efficacy in our initial experience according to previously published results. In some centers, a 35 mm diameter catheter has been used for larger veins [4, 5, 6]; however, in our study, it appears that a 31 mm catheter is sufficient for PVI in all the patients.

The rate of incomplete PV isolation with the first standard applications is low (5%) and it mainly affects the anterior segments of the pulmonary veins.

Bohnen et al. [1] described that more than 50% of patients showed an insufficient ablation area, mainly in the anterior aspect of the left pulmonary veins. This is in contrast with our results. In our series, only 23% of patients showed a notch of healthy tissue in the anterior carina, mainly in the right pulmonary veins. This area is near the transeptal puncture and may be difficult to position the catheter properly. The anterior aspect of the right pulmonary veins is also the most common gap found in patients without first‐pass PV isolation in our study.

Bohnen et al. also found that 26% of patients had PV isolation lines fusion at the posterior wall or at the roof level. Our study confirms a similar incidence using the Rhythmia system. 23% of patients showed fusion at the posterior wall when the voltage map was analyzed. As additional data, we found that 2 out of 18 patients with fusion in the posterior wall did not have conduction block but rather persistence of conduction through it with slow velocity when the activation map was analyzed. This combined with the finding that patients with a very narrow corridor (< 10 mm) on the posterior wall had slower conduction velocity through it could be the substrate for the maintenance of atypical atrial flutter after PVI. In our series, 1 out of 3 with post‐PVI atypical atrial flutter showed a very narrow corridor with slow velocity in the post‐PVI map.

If the excessive posterior wall ablation using PFA has an impact on the clinical results is uncertain. Urbanek et al. [2] report a high incidence of atypical atrial flutter after Farapulse PFA ablation (16%) versus cryoballon (7.5%) including the blanking period. The examination of the mechanism of the atypical flutter revealed that a posterior wall–dependent atrial flutter was present in 5 of 10 patients in whom the mechanism could be analyzed. The critical isthmus was found to be at the posterior wall/roof between the lesions created for PVI during the index procedure [2]. In contrast, our series only showed 3.6% of patients with atypical atrial flutter during follow‐up, including the blaking period. Only 1 of 3 patients with atypical atrial flutter had showed a narrow corridor with slow activation through it in the post‐ablation map.

The potential explanations for the posterior wall unexpected ablation involvement are as follows: (1) The catheter is navigated mainly by fluoroscopic guidance and may have an unnoticed position that is slightly too posterior. (2) The catheter may be oversized for a small atrium. In our experience, the size of the left atrium is important to predict posterior wall ablation involvement. An inter‐carina distance of < 65 mm predicted either a narrow aisle or a circumferential PV lines fusion at the posterior wall.

The difference found between Urbanek's series and ours could be explained by the different use of the 31 and 35 mm diameter catheters. Urbanek et al. used both catheter sizes [2, 5]. It could have led to a more frequent formation of large lesions and a frequent presence of narrow corridors in the posterior wall that serve as the substrate for the flutter maintenance. In our study, only the 31 mm diameter catheter was used.

Whether it is necessary to reinforce, with additional applications, the unnoticed ablation fusion or narrow corridor at the posterior wall to avoid slow velocity corridors is unknown.

Finally, the previous knowledge of atrial size itself could be important in the catheter selection (31 mm vs. 35 mm) and the catheter positioning to avoid excessive ablation on the posterior wall. A future complete integration of this catheter with a navigation system will also allow better control of the catheter positioning.

## Author Contributions

All authors contributed to the design, data analysis, critical revision of article, approval of article, statistics, and data collection.

## Ethics Statement

Ethical approval was waived by the local Institutional Review Board in view of the retrospective nature of this study. The study confirms with the principles of the Declaration of Helsinki.

## Conflicts of Interest

The authors declare no conflicts of interest.

## Data Availability

The authors have nothing to report.

## References

[1] M. Bohnen , R. Weber , J. Minners , et al., “Characterization of Circumferential Antral Pulmonary Vein Isolation Areas Resulting From Pulsed‐Field Catheter Ablation,” Europace 25 (2023): 65–73. 35852306 10.1093/europace/euac111 PMC10103571

[2] L. Urbanek , S. Bordignon , D. Schaack , et al., “Pulsed Field Versus Cryoballoon Pulmonary Vein Isolation for Atrial Fibrillation: Efficacy, Safety, and Long‐Term Follow‐Up in a 400‐Patient Cohort,” Circulation. Arrhythmia and Electrophysiology 16 (2023): 389–398. 37254781 10.1161/CIRCEP.123.011920

[3] A. Verma , L. Boersma , D. E. Haines , et al., “First‐in‐Human Experience and Acute Procedural Outcomes Using a Novel Pulsed Field Ablation System: The PULSED AF Pilot Trial,” Circulation. Arrhythmia and Electrophysiology 15 (2022): e010168. 34964367 10.1161/CIRCEP.121.010168 PMC8772438

[4] B. Schmidt , S. Bordignon , S. Tohoku , et al., “5S Study: Safe and Simple Single Shot Pulmonary Vein Isolation With Pulsed Field Ablation Using Sedation,” Circulation. Arrhythmia and Electrophysiology 15 (2022): e010817. 35617232 10.1161/CIRCEP.121.010817

[5] V. Y. Reddy , E. P. Gerstenfeld , A. Natale , et al., “Pulsed Field or Conventional Thermal Ablation for Paroxysmal Atrial Fibrillation,” New England Journal of Medicine 389 (2023): 1660–1671, 10.1056/NEJMoa2307291. 37634148

[6] E. Ekanem , V. Y. Reddy , B. Schmidt , et al., “Multi‐National Survey on the Methods, Efficacy, and Safety on the Post‐Approval Clinical Use of Pulsed Field Ablation (MANIFEST‐PF),” Europace 24 (2022): 1256–1266. 35647644 10.1093/europace/euac050 PMC9435639

